# Microstructure Evolution in Mg-Zn-Zr-Gd Biodegradable Alloy: The Decisive Bridge Between Extrusion Temperature and Performance

**DOI:** 10.3389/fchem.2018.00071

**Published:** 2018-03-20

**Authors:** Huai Yao, Jiu-Ba Wen, Yi Xiong, Yan Lu, Marko Huttula

**Affiliations:** ^1^School of Materials Science and Engineering, Henan University of Science and Technology, Luoyang Henan, China; ^2^Collaborative Innovation Center of Nonferrous Metals of Henan Province, Luoyang Henan, China; ^3^Nano and Molecular Systems Research Unit, University of Oulu, Oulu, Finland

**Keywords:** magnesium alloy, microstructure, dynamic recrystallization, extrusion, corrosion

## Abstract

Being a biocompatible metal with similar mechanical properties as bones, magnesium bears both biodegradability suitable for bone substitution and chemical reactivity detrimental in bio-ambiences. To benefit its biomaterial applications, we developed Mg-2.0Zn-0.5Zr-3.0Gd (wt%) alloy through hot extrusion and tailored its biodegradability by just varying the extrusion temperatures during alloy preparations. The as-cast alloy is composed of the α-Mg matrix, a network of the fish-bone shaped and ellipsoidal (Mg, Zn)_3_Gd phase, and a lamellar long period stacking ordered phase. Surface content of dynamically recrystallized (DRXed) and large deformed grains increases within 330–350°C of the extrusion temperature, and decreases within 350–370°C. Sample second phase contains the (Mg, Zn)_3_Gd nano-rods parallel to the extrusion direction, and Mg_2_Zn_11_ nanoprecipitation when temperature tuned above 350°C. Refining microstructures leads to different anticorrosive ability of the alloys as given by immersion and electrochemical corrosion tests in the simulated body fluids. The sample extruded at 350°C owns the best anticorrosive ability thanks to structural impacts where large DRXed portions and uniform nanosized grains reduce chemical potentials among composites, and passivate the extruded surfaces. Besides materials applications, the *in vitro* mechanism revealed here is hoped to inspire similar researches in biometal developments.

## Introduction

The magnesium (Mg) is an essential element for human metabolism where excess amount of the element is harmlessly excreted in urine (Hermawan et al., 2010; Bakhsheshi-Rad et al., 2014; Chen et al., 2014). The non-toxic metal has similar mechanical properties as bones in stress release (Nagels et al., 2003), enabling its potentials to help bone recovery after implantation (Staiger et al., 2006). To extend its usability, the Mg has further been alloyed with different metals and biodegradable implants have been reached (Witte et al., 2006; Witte, 2010; Jafari et al., 2015). Recent investigations further show that the stabilities and durability of the metal and its alloys can be switched in at different ambiences, provided dedicated materials engineering is performed for the structural and compositional treatments (Jamesh et al., 2014; Mareci et al., 2016).

Despite the biocompatibility and mechanical uniqueness, the Mg and its alloys suffer from robust chemical reactivity in aqueous environment where hydrogen is released following Mg and H_2_O reaction. This rapid corrosion rate results in loss of mechanical strength of the implants before accomplishing their purposes (Wang et al., 2012; Ding et al., 2014). Alloying it with proper metals may enhance its anticorrosive performances. For instance, Zn can transform impurities, such as Fe and Ni, into harmless intermetallic compounds and improve the corrosion resistance of Mg alloys at a content below 5wt.% (Rosalbino et al., 2010). Though with a relatively low solubility in the Mg matrix, small amount of Zr addition can substantially improve the corrosion performances of Mg alloys (Gu et al., 2011). Besides improving mechanical properties (Zhang et al., 2012a), introduction of rare earth elements (REE) into magnesium alloys is also an effective way to enhance corrosion resistance of the magnesium alloys (Song and StJohn, 2002; Gui et al., 2016). Similar strengthening effects can also be achieved by using the Gd as an alloy metal. Research evidences also proved that many REEs are anti-carcinogenic (Dai et al., 2002; Feyerabend et al., 2010). The Gd also possesses the property, and can diminish the amount of cancer cells in the studies (Magda and Miller, 2006; Kostova et al., 2007). Unfortunately, free Gd^3+^ ion is toxic. Though it can be excreted slowly from human body through metabolism, the accumulation of excessive Gd^3+^ will induce apoptosis of cells in a short term. Thus, it is necessary to lower the degradation rate of the Gd element to the median lethal dose level (LD50) in the body to ensure the biosafety (Ersoy and Rybicki, 2007). Combining the Zn, Zr, and REs to the Mg matrix, the Mg-Zn-Zr-X (X = RE) quaternary alloys have further been developed. While mechanical strengths have been explored, corrosion tests were performed through hydrogen evolution experiment (Zhang et al., 2012b). Besides the rather complicated synthesis, it is still unclear for the alloy performances and preparation conditions influences on the performances in bio-ambiences.

In this work, we employed hot extrusion route to prepare the Mg-2.0Zn-0.5Zr-3.0Gd (wt%) biometallic alloys, and studied extrusion temperature impacts on their microstructures and anticorrosive performances in the simulated body fluid (SBF). Benefiting from the hot preparation route at the optimized temperature, dynamic recrystallization (DRX) leads to grain refinements of the alloys. Consequentially, mechanical deformations are enlarged, and the alloys are more resistant in simulated bio-ambiences. The *in vitro* origin for materials functionality improvements is also proposed.

## Experimental procedure

**Figure 1:**
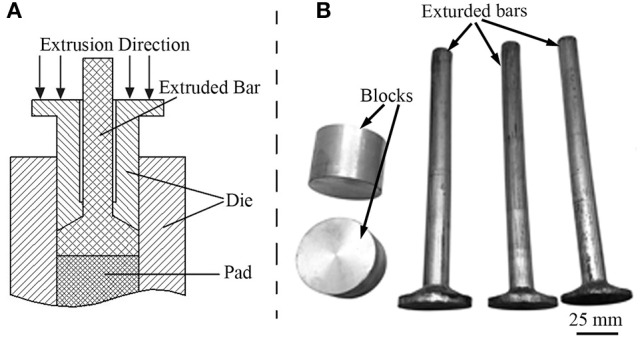
**(A)** Schematic illustration of the extrusion; **(B)** Blocks and extruded bars.

Alloy ingot with an actual composition of Mg-2.0Zn-0.5Zr-3.0Gd alloy (wt%) was melted from high purity Mg (purity ≥ 99.93%) and Zn (purity ≥ 99.93%) ingots. The Mg-25Zr(wt%) and Mg-20Gd(wt%) master alloys were processed under the protective CO_2_+1%SF_6_ gas mixture in order to prevent burning of the Mg in an electronic induction furnace. The equivalent amount of alloy was added when the temperature reached about 730°C. The melt alloys were further held at 730 °C for ~20 min and then poured into a preheated mild steel mold of 160 × 45 × 100 mm in size. As schematic illustration of the extrusion and extruded bars are shown in Figure 1. The cast ingots were machined into blocks with a size of Φ50 × 35 mm for extrusion. The cylindrical ingots were extruded at 330, 340, 350, 360, and 370°C, and the temperature was measured by a pyrometer infrared thermometer (DIAS, Germany). They are labeled as E330, E340, E350, E360, E370, respectively. Then the cylindrical ingots were extruded into rods with a diameter of 18 mm, an extrusion ratio of ~8 at a speed of 5 mm/s. The extruded rods were naturally cooled to room temperature, and annealed 4 h at 200°C afterwards.

The specimens for microstructure observations were cut parallel to the extrusion direction (ED) and mechanically polished. Afterwards, they were etched with the 0.19 mol/L picric acid solutions which is obtained by dissolving 5 g picric acid in 5 mL acetic acid, and successively mixed with 100 mL absolute ethyl alcohol and 10 mL distilled water. The microstructure of the specimens at different conditions was observed using optical microscopy (OLYMPUS) and scanning electronic microscopy (SEM, JSM-5610LV) in back-scattered electron (BSE) mode. By counting the characteristic X-ray emissions through the energy dispersive spectroscopy (EDS), the SEM can further make element identification locally. X-ray diffraction (XRD) experiments were carried out using the D8 ADVANCE X-ray diffractometer. The tube voltage and current were 35 kV and 40 mA, respectively. The scan rate was 0.02°/s with 1 s per step. The detailed microstructure of the extruded samples was examined using transmission electron microscopy (TEM, JEM-2010) operated at 200 kV. The TEM samples were machined by wire-cutting the rods parallel to ED, and then treated by fluid jet polishing under the 97% ethyl alcohol and 3% perchloric acid mixture. Finally, they were iron milled using the twin gun precision ion polishing system (Gatan, model 691).

Anticorrosive performances of the alloys were carried out in SBF which has chemical composites and concentrations as tabulated in Table 1 (Jeong and Kim, 2014). Evaluations of anticorrosion were performed through two experiment sequences of immersion corrosion test and electrochemical corrosion test. In the first one, samples with a diameter of 18 mm and a thickness of 5 mm were immersed in 180 mL solutions and the temperature was maintained at 37 °C under a water bath. The immersion lasted 120 h during which the SBF was renewed every 8 h in order to keep a relative stable pH value. An electrochemical workstation (Autolab PGSTAT128N) was employed in the electrochemical test, and a three-electrode configuration was adopted. The alloy sample (Φ11.3 × 10 mm) was used as working electrode, a saturated calomel electrode as reference electrode, and a graphite sheet as counter electrode. The working electrodes were connected to a copper wire and then embedded in the epoxy resin. The mounted samples were mechanically polished and exposed a surface area of 1 cm^2^. Potentiodynamic polarization testing was performed at a scanning rate of 1 mVs^−1^ from −0.25 V in the cathodic direction to +0.4 V in the anodic direction based on the open circuit potential.

**Table 1:** Composition of SBF solution.

**Component**	**Concentration/(g·L^−1^)**
NaCl	8.00
CaCl_2_	0.14
KCl	0.40
NaHCO_3_	0.35
MgCl_2_·6H_2_O	0.10
Glucose	1.00
Na_2_HPO_4_·12H_2_O	0.06
KH_2_PO_4_	0.06
MgSO_4_·7H_2_O	0.06

## Results and discussion

### Microstructure and phase analysis of as-cast alloys

**Figure 2:**
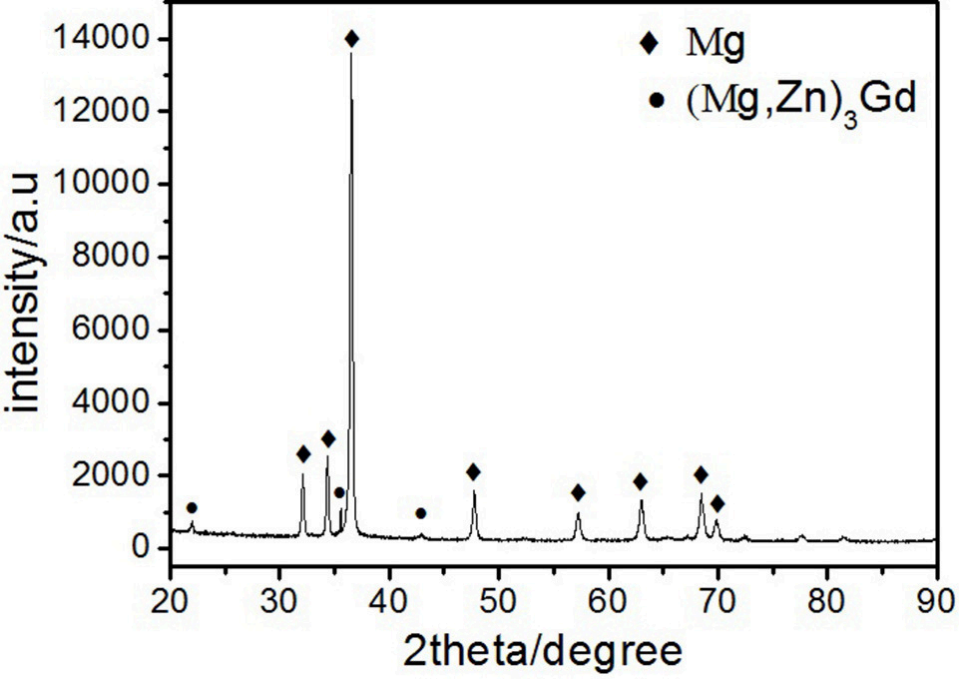
XRD patterns of the as-cast Mg-2.0Zn-0.5Zr-3.0Gd alloy.

**Figure 3:**
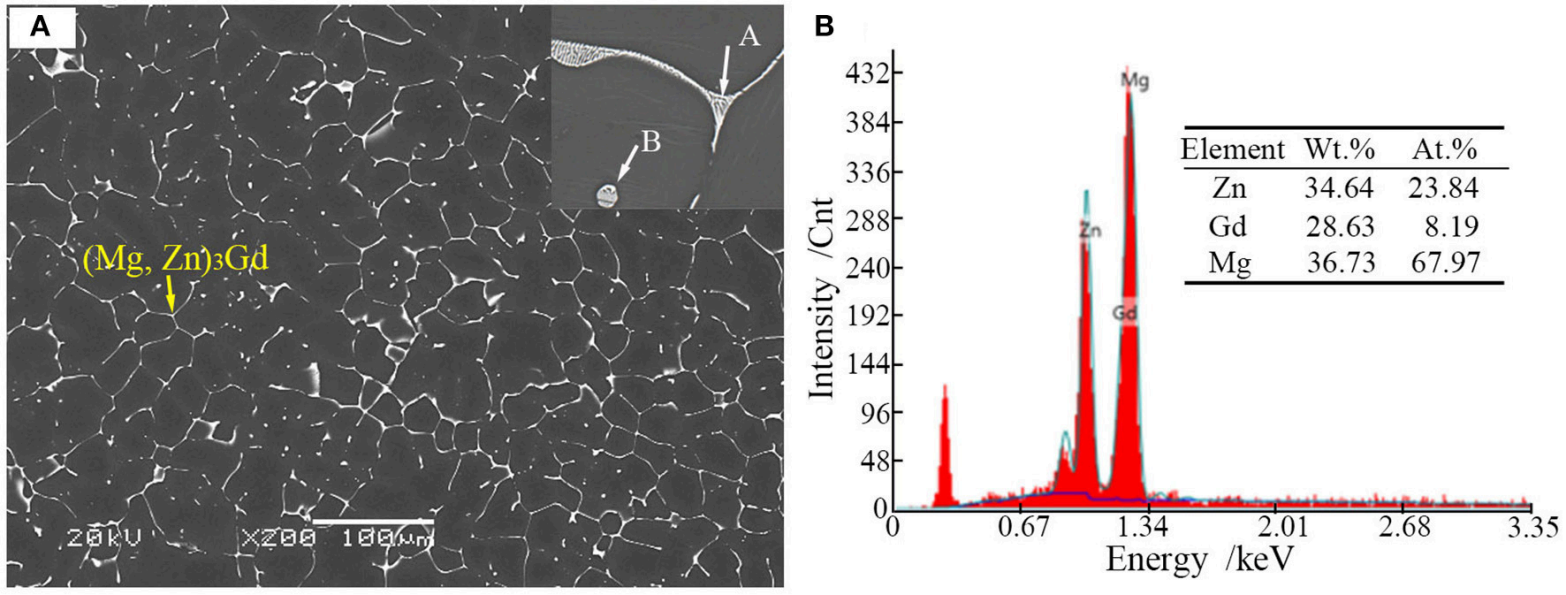
**(A)** Scanning election microscopic image and enlarged image (onset) of the as-cast Mg-2.0Zn-0.5Zr-3.0Gd alloy; **(B)** EDS analysis results of the eutectic compounds.

Microstructures of the as-cast alloys were firstly alloyed. As depicted in Figure 2, sharp peaks in the XRD pattern are well indexed to α-Mg matrix (JCPDS89-5003) and (Mg, Zn)_3_Gd (JCPDS 65-0040) (Zhang et al., 2016). No other peaks can be observed in the XRD, indicating the purity of the as-prepared samples. Figure 3A shows the SEM image of the as-cast Mg-2.0Zn-0.5Zr-3.0Gd alloy. The black α-Mg matrix is decorated with a network of the fish-bone shaped (marked as “A”) and ellipsoidal eutectic compounds in Figure 3A. In contrast to these compounds at grain boundaries, the small ellipsoidal shaped eutectic phases (marked as “B”) are distributed across the interior of the grains. The EDS analysis of the fish-bones shows the existences of characteristic lines from only Mg, Zn, and Gd in Figure 3B. The ellipsoidal and fish-bone shaped eutectic compounds have similar element composition. These are consistent with the XRD results, where the phase composition in the as-cast alloy is identified as α-Mg and (Mg, Zn)_3_Gd phases.

**Figure 4:**
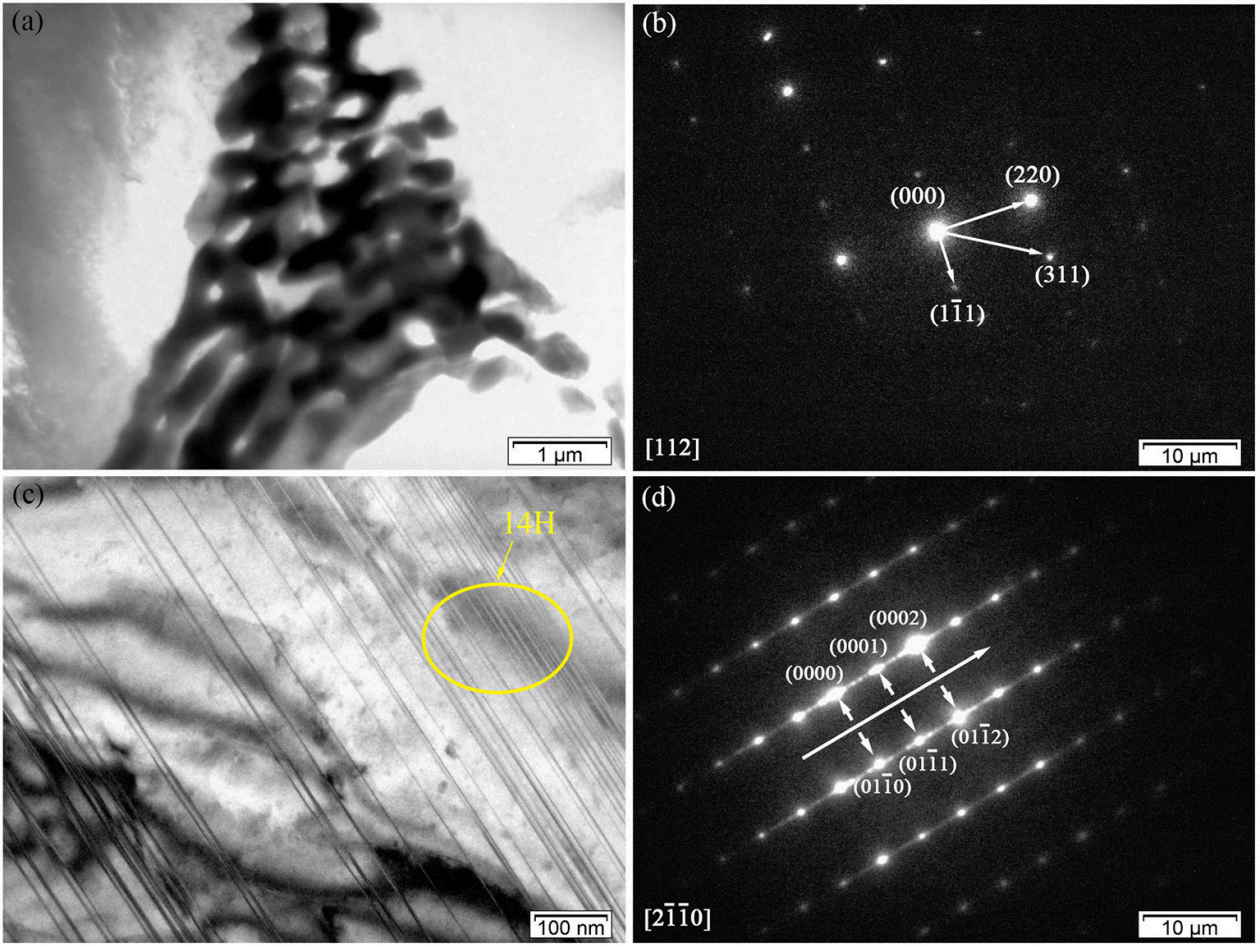
Microstructure of the as-cast Mg-2.0Zn-0.5Zr-3.0Gd alloy: **(a)** BF TEM image of continuous network eutectic phase; **(b)** SAED pattern corresponding to the eutectic phase; **(c)** BF TEM image of the LPSO phase distributed inside the grains; **(d)** SAED pattern corresponding to the LPSO phase.

Figures 4A,B shows the TEM bright-field (BF) image of the fish-bone shaped eutectic phase at the grain boundary in the as-cast Mg-2.0Zn-0.5Zr-3.0Gd alloy, and its corresponding selected area electron diffraction (SAED) pattern along the $\left[\bar{1}12\right]$ direction. According to the SAED pattern, the phase has face-centered cubic (fcc) structure with a lattice constant of 0.729 nm. Thus, this phase was identified as the Mg_3_Gd phase. However, the EDS results (Figure 3B) reveal the existence of a small quantity of Zn, similar to results given by Srinivasan (Srinivasan et al., 2014). It can be ascribed to solid solution based on the Mg_3_Gd, and expressed as (Mg, Zn)_3_Gd. Moreover, the Zn and Gd content variations lead to small changes in the lattice parameters of the (Mg, Zn)_3_Gd phases (Xu et al., 2013). The Zn and Mg contents in the (Mg, Zn)_3_Gd differ from the original values at the sample location in the as-cast Mg-2.0Zn-0.5Zr-3.0Gd alloy. The lattice parameter of the (Mg, Zn)_3_Gd phases significantly decrease with the Zn/Mg ratio in the (Mg, Zn)_3_Gd.

Figures 4C,D show the BF images of the fine lamella long-period stacking ordered (LPSO) phase in the matrix, and corresponding SAED pattern recorded along the ${\left[2\bar{1}\bar{1}0\right]}_{\alpha -Mg}$ direction. Located at the positions of n/7, where n is an integer of the (0002)_α−*Mg*_ diffraction, the bright points verify the 14H type for the LPSO (Xu et al., 2013). The emerging of LPSO phase may be due to solid reactions between (Gd, Zn) elements with proper content ratio and condition in forming the 14H phase. The low concentration of solute atoms could lead to regular dislocations in the Mg matrix. The compression of solidly solved atoms also results in the decrease of lattice spacing along the c-axis.

### Extrusion temperature impact on microstructures

**Figure 5:**
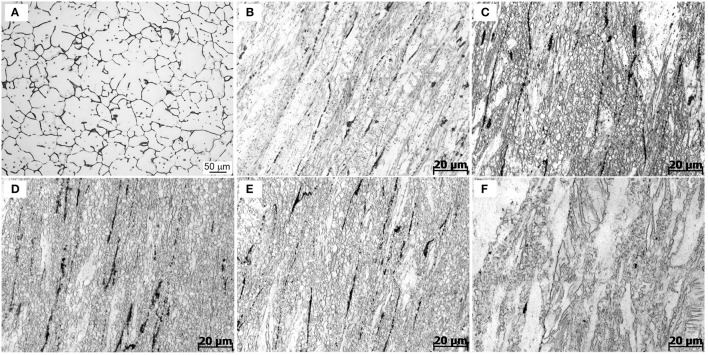
Optical images of the Mg-2.0Zn-0.5Zr-3.0Gd alloys: **(A)** As-cast;**(B)** E330; **(C)** E340; **(D)** E350; **(E)** E360; and **(F)** E370.

This subsection presents and discusses the influences of extrusion temperature on microstructures of the extruded samples. Figure 5 shows the optical micrographs of as-cast and extruded alloys along the longitudinal sections. As-cast alloy exhibits equiaxed microstructure with a grain size of about 25 μm, as seen in Figure 5A. Figures 5B–F show the optical micrographs of the Mg-2.0Zn-0.5Zr-3.0Gd alloy at various extrusion temperatures. Compared with the microstructure of the as-cast alloy, the whole extruded matrix was nearly bimodal-grained along the extrusion direction, which contains both the non-dynamic recrystallized (unDRXed) deformed grains and fine DRXed grains. Large deformed grains have strong basal texture and fine recrystallized grains own weaker texture. The bimodal-grained microstructure is commonly observed in extruded Mg alloys (Du et al., 2015). The recrystallization starts from the grain boundary of the former grains and the secondary phase, while the elongated grains are due to the extrusion (Homma et al., 2010). It could be seen that the area fraction of DRXed microstructure increased with extrusion temperature within the 330–350°C range, followed by a decrease at 350–370°C. The area fraction of DRXed microstructure and grain size of E350 are estimated to be about 95% and 5 μm, respectively. The cast microstructure of the Mg-2.0Zn-0.5Zr-3.0Gd alloy after extrusion completely disappeared. The grain size of the alloy was obviously refined, and the microstructure in the interior of the alloys had no cracks nor pores. The continuous secondary phase was mainly changed into black parallel strips distributed along the extrusion direction. The secondary phase has been plastically deformed during extrusion, which acted as the preferred DRXed nucleation sites and stimulated nucleation of the DRXed grains in Figures 5C–E (Robson et al., 2009). Furthermore, stress concentration occurred near secondary phases, resulting in dislocations piling up. These accumulations promoted the nucleation of recrystallized grains and enhanced the DRX during the extrusion process. When infused with REs, the Mg alloys turn to have finer recrystallized grains during extrusion and rolling processes (Luo et al., 2014).

Remarkably in Figures 5C–E, many DRXed grains are not only evolved at the original grain boundaries, but also inside of grains. The experimental evidences indicate that with increasing extrusion temperature, more slip systems are activated and the effects of plastic anisotropy are weaker. This results in the occurrence of a more homogeneous deformation and simultaneously the increase of the DRX ratio (Zhang et al., 2015). The DRX partly occurred in the extrusion process, while the grains were simultaneously refined. The large shear force caused the alloy to break and distribute in the matrix.

The temperature has a prominent role to determine the extrusion microstructures. The actual extrusion temperature is not the same as the initial sample temperature due to the heat generation from deformation heat, friction heat, and heat dissipation to the surroundings. Increasing temperature could further refine the grains to be more uniform in the 330–350°C range. When the temperature was set to 330°C, the DRX was not evolved. The rather coarse grains are not uniform in size. When the extrusion temperature was increased to 350°C, the grain size decreased significantly and became more uniform. More grain nucleation happened at higher temperature. This is due to a large quantity of plastic deformation when the extrusion ratio is nearly 8, which provides favorable conditions for the recrystallization. The nucleation of DRX also requires a critical temperature of deformation, and can occur when the actual deformation temperature reaches a critical temperature. The secondary phase, with a relatively high melting point, can pin grain boundaries and hindered grain growth during hot extrusion. In this case, the change of grain shapes is not obvious, as the extrusion temperature increases from 340 to 360°C. However, when the extrusion temperature reaches 370°C, the thermal stability of the secondary phase decreases, and the pinning effect of particles is rapidly weakened. Therefore, a significant grain coarsening occurs at 370°C. Thus, recrystallization depends on both temperature and extrusion ratio, and the temperature determines the nucleation rate. The amount of grain growth is controlled by temperature at certain extrusion ratios.

**Figure 6:**
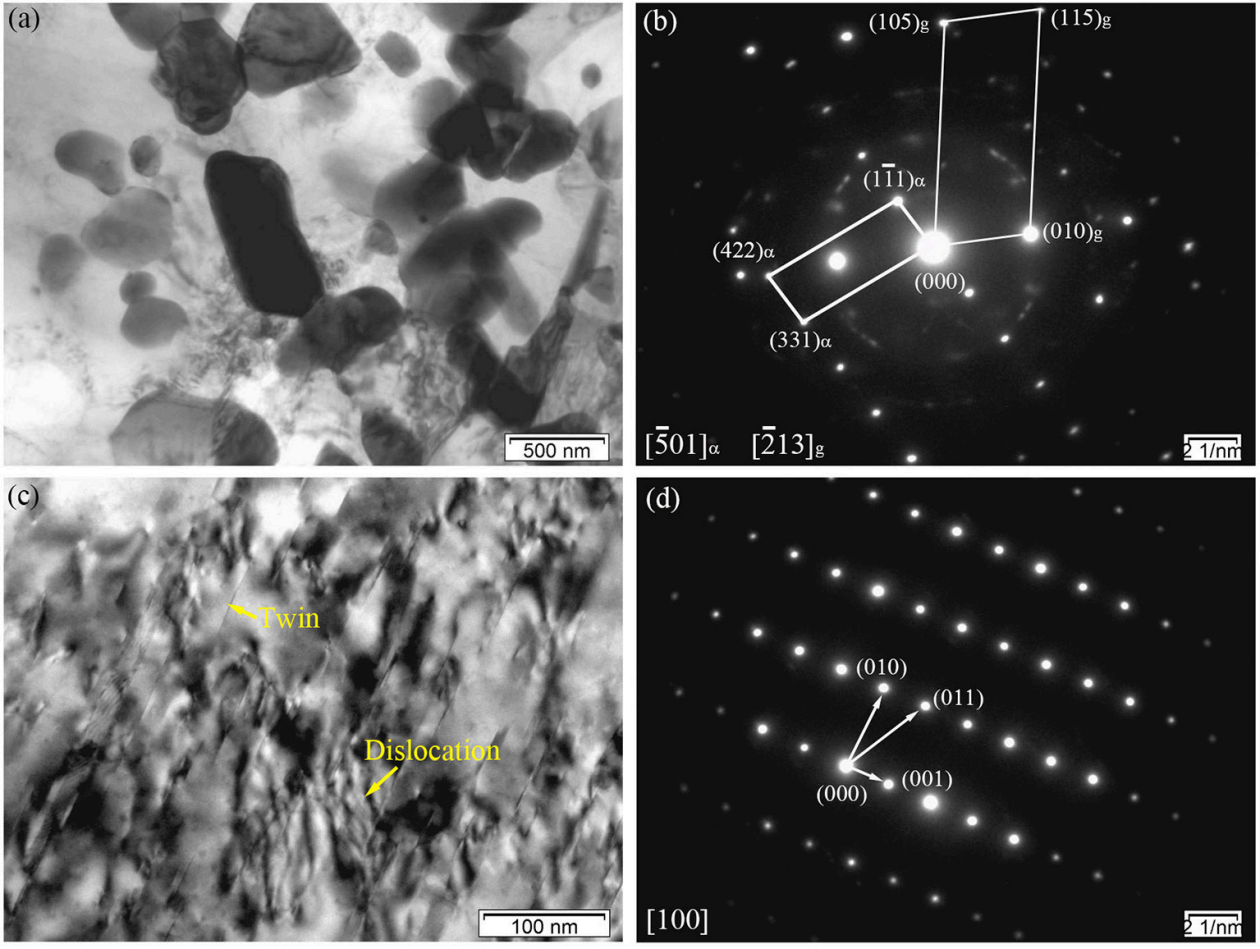
Microstructure of the E330 sample: **(a,c)** BF images; **(b)** Corresponding SAED pattern of **(a)**; **(d)** Corresponding SAED pattern of **(c)**.

Figure 6a shows the BF image of the particles with a sub-micrometer size distributed at the grain boundaries in the E330 sample. It can also be speculated that large sized secondary phase grains existed in the as-cast alloy and were broken into smaller ones. Two kinds of lattice structures were observed in the SAED patterns in Figure 6b. One was hexagonal close packed (hcp) lattice of α-Mg, and another the particle fcc phase. In the particle phase of the hot extruded alloy, the SAED pattern was similar to that of the as-cast alloy with fcc signature. With a calculated lattice parameter of ~0.721 nm, the hot extruded alloy was identified as (Mg, Zn)_3_Gd, similar to those observed in the as-cast alloy (see Figure 4). However, this number is smaller than 0.729 nm of the as-cast, indicating more dissolution of Zn into the (Mg, Zn)_3_Gd phase from the α-Mg matrix after hot extrusion. As shown in Figure 6c, twins and dislocations are observed within the grains. The LPSO phases also turn up here, similar to the observation in Figure 4c. However, instead of LPSO phase, a large number of twin crystals was found inside the grains. The corresponding SAED patterns shown in Figure 6d further prove the twins are α-Mg. We attribute the origin as follows. Typically the dislocations are generated during hot extrusion. In addition, dislocations pile-up at twin boundaries, which could induce sub-grain formation and contribute to recrystallization (Sun et al., 2016). Deformation twinning is an important mechanism in compressive stress along the c-axis, while twin crystals facilitate deformation and increase plasticity.

**Figure 7:**
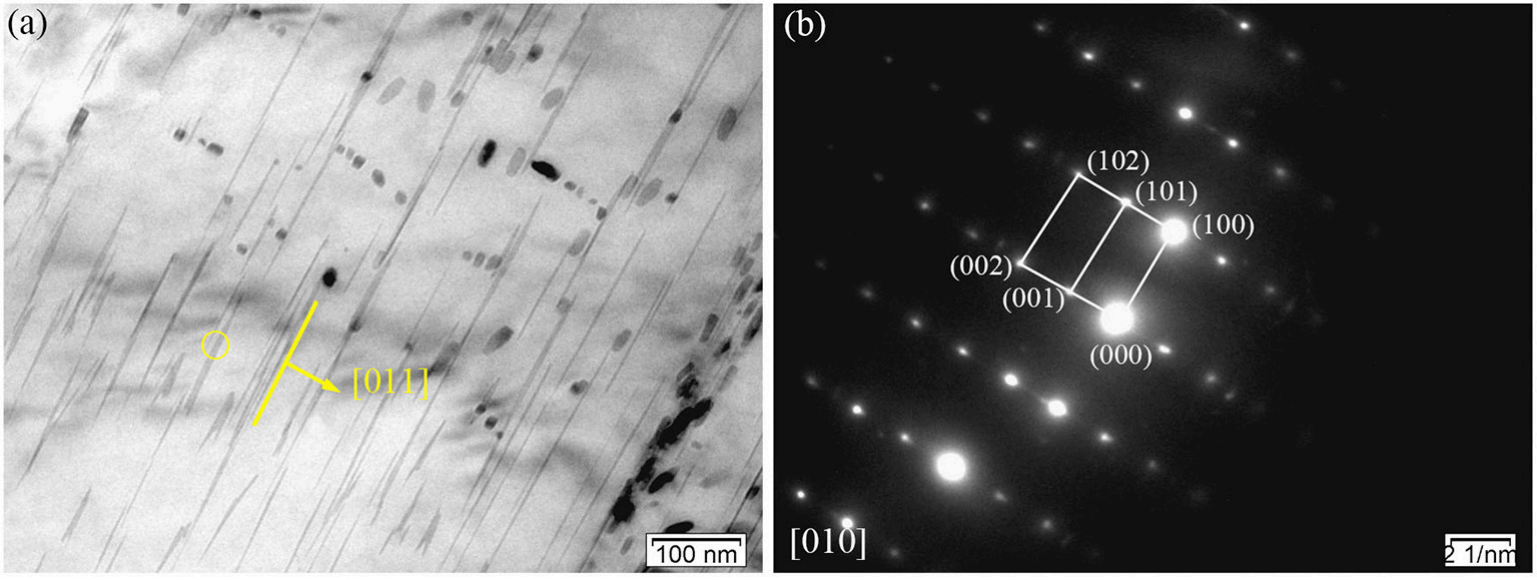
Microstructure of the E350 sample: **(a)** BF image; **(b)** Corresponding SAED pattern.

With increased extrusion temperature, the activation of non-basal slip systems is more likely to occur since the value of critical resolved shear stress decreases rapidly with temperature. This can be seen in Figures 7a,b, where the twinning deformation of the E350 sample decreased compared to E330. In addition, the activation of twinning is suppressed with extrusion temperature, and a transition from twin-dominated to slip-dominated deformation may occur (Huang and Log, 2016). A small quantity of deformation twins exist in the α-Mg matrix far from the secondary phase.

**Figure 8:**
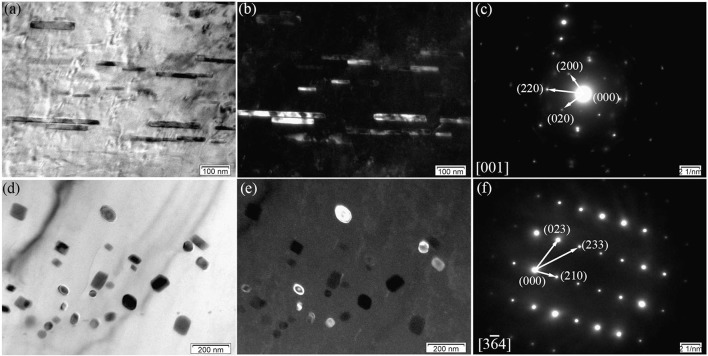
Microstructure of the E350 sample; **(a,b)** BF and dark-field micrographs of the rod-like precipitates; **(c)** Corresponding SAED pattern of **(a,b)**; **(d,e)** BF and dark-field micrographs of the grain-like precipitates; **(f)** Corresponding SAED pattern of **(d,e)**.

Figure 8 displays the TEM micrographs of the E350 sample. In Figure 8a, the BF image shows loosely dispersed rod-like precipitates with 100–400 nm length and 20 nm width. The dark-field image in Figure 8b shows rod-like precipitates oriented along the ED. According to the SAED pattern in Figure 8c, the rod-like precipitated phase was also found to have an fcc structure with a = 0.722 nm. Hence, this rod-like precipitated phase was confirmed to have (Mg, Zn)_3_Gd phase, similar to the Mg_3_Gd type with Zn dissolved in the structure.

In addition, some of nanoscale elliptical particles stay within α-Mg matrix, as seen in Figures 8d,e. The average size of the elliptical particles is within 5–20 nm range. According to the SAED pattern in Figure 8f, the elliptical particles have a primitive cubic lattice structure with a = 0.865 nm. Therefore, these elliptical particles are suggested to have the Mg_2_Zn_11_ phase with their axis parallel to the $\left[3\bar{6}4\right]$ direction. Noticing the absence in as-cast alloys, the Mg_2_Zn_11_ phase is formed only after extrusion in the Mg-2.0Zn-0.5Zr-3.0Gd alloy. This may be due to the redistribution of Zn element in the matrix at high temperatures, which precipitated as the Mg_2_Zn_11_ phase during the extrusion process.

As illustrated in Figures 8a–f, these nanoscale rod-like and elliptical particles tend to precipitate in the DRXed areas rather than the original precipitated phase breakage. This means that a high deformation temperature can cause the partial dissolution of pre-existing (Mg, Zn)_3_Gd precipitates. Some nanoscale rod-like and elliptical particles dispersed in the matrix at a temperature range of 340–360°C. Thus, dynamic precipitation can also occur during extrusion at appropriate deformation temperatures, and yield many nanoscale precipitated phases in the extruded alloy.

**Figure 9:**
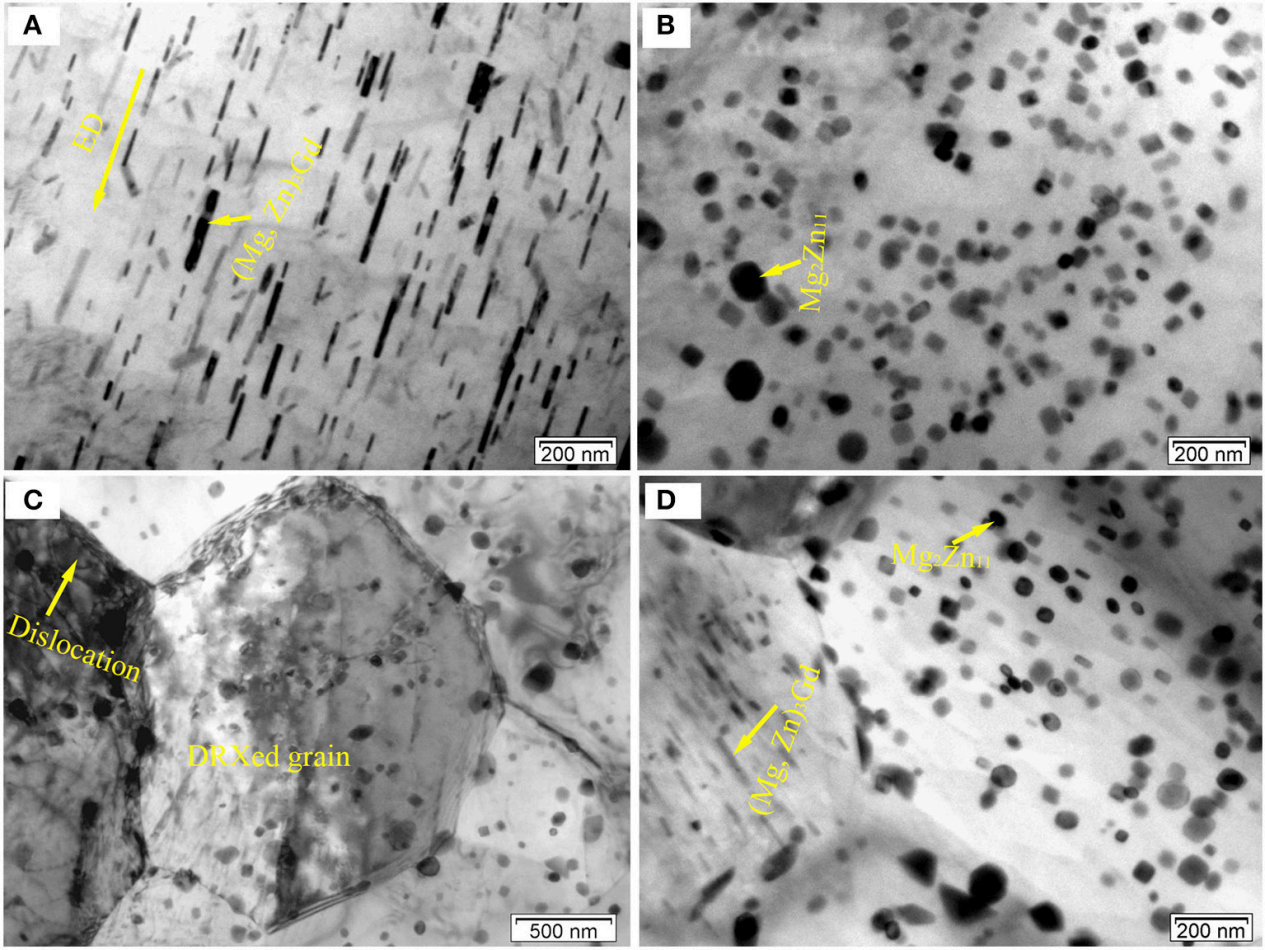
Typical TEM BF images of the E350 samples: **(A)** A large amount of rod-like precipitates; **(B)** A large amount of granular precipitates; **(C,D)** Rod-like and granular precipitates at the grain boundaries or within crystals.

Figure 9 shows typical TEM micrograph of the E350 samples. In Figure 9A, an intensive dispersion of rod-like precipitates are found parallel to ED. They own the (Mg, Zn)_3_Gd phase, according to previous determination. The rod-like form of the (Mg, Zn)_3_Gd phase can be seen as the dissolution and re-precipitation of the pre-existing (Mg, Zn)_3_Gd phase during hot extrusion. A random distribution of granular precipitates in the α-Mg matrix is obvious in Figure 9B. The granular form of Mg_2_Zn_11_ (Figure 8F) becomes a newly precipitated phase in the extrusion process. A large number of Mg_2_Zn_11_ phase particles exist in the DRXed region. They have the grain size of 10–30 nm.

From Figures 9C,D, these precipitates distribute heterogeneously within DRXed grains and some boundaries of DRXed grains. In addition, some dislocations can be observed within the grains. It should be noted that the size of the DRXed grains is about 2–6 μm. These precipitates can form during extrusion and influence the recrystallization behavior. As for the recrystallization behavior, the precipitates can act as nucleation sites during the process, and may also exert a significant pinning effect on the grain boundaries. Indeed, precipitates can either promote or suppress recrystallization, depending on their morphologies such as size, shape, volume fraction, and spacing (Sun et al., 2016). As seen in Figures 9C,D, the Mg_2_Zn_11_ phase particles, with the 20–50 nm size, and rod-like form of (Mg, Zn)_3_Gd phase, with the 50–200 nm size in length and 20 nm in width, mainly occur in the DRXed region. Fine secondary phase particles tend to hinder boundary motion by slowing down recrystallization and grain growth. In contrast, coarse secondary phase particles can accelerate recrystallization by particle-stimulated nucleation due to the large amount of stored energy in the deformation zones (Hou et al., 2011). This means that these (Mg, Zn)_3_Gd and Mg_2_Zn_11_ phase particles, with sizes far less than 1 μm, inhibit grain boundary migration. Therefore, these fine secondary phase particles may contribute to grain refinement via grain boundary pinning.

### Anti-corrosive tests

The anticorrosion results in the immersion test is evaluated through weight loss rate Δ*W*(mg/cm^2^/d). This can be translated to an average corrosion rate (mm/y) using

(1) $$\begin{array}{r} P_{w}=3.65\Delta W/\rho \end{array}$$

**Figure 10:**
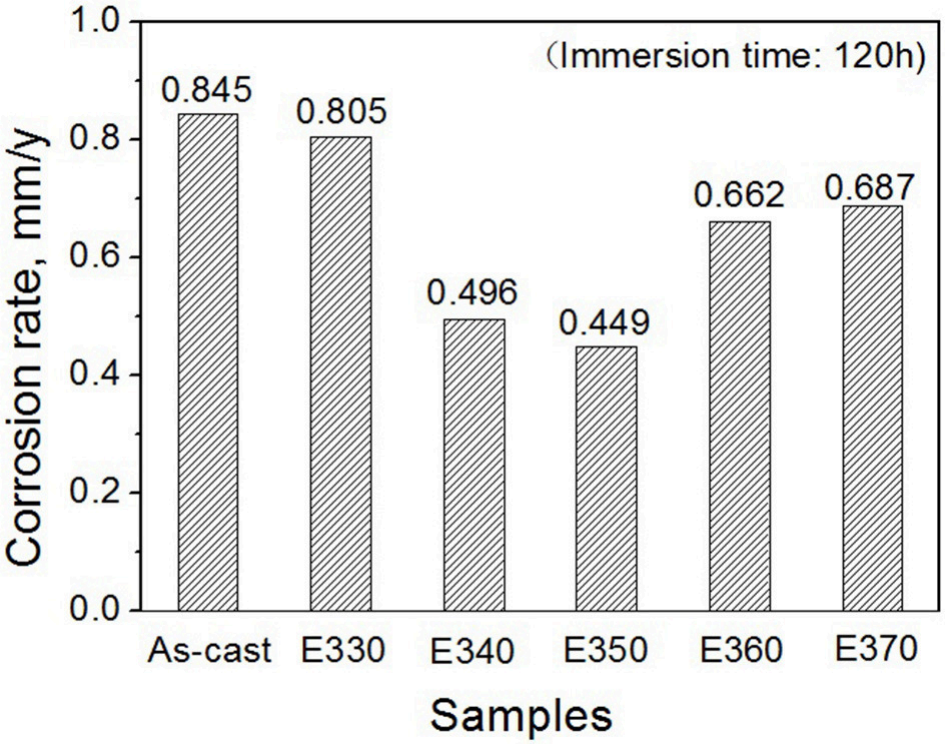
Corrosion rates for Mg-2.0Zn-0.5Zr-3.0Gd alloy measured by weight loss after immersed for 120 h.

**Figure 11:**
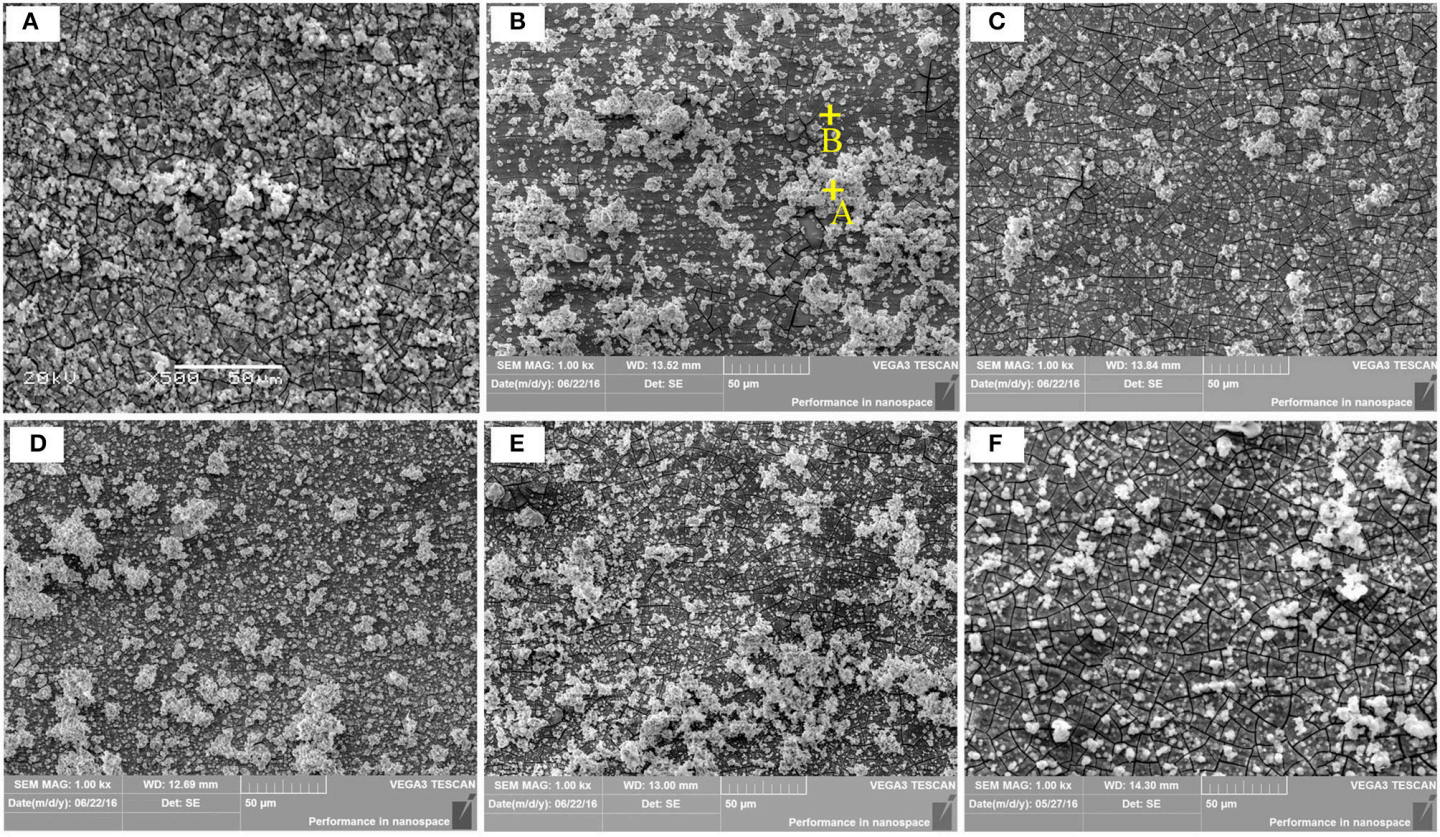
Corrosion micrographs of the as-cast Mg-2.0Zn-0.5Zr-3.0Gd alloys after immersion in SBF for 120 h. **(A)** As-cast; **(B)** E330; **(C)** E340; **(D)** E350; **(E)** E360; **(F)** E370.

**Figure 12:**
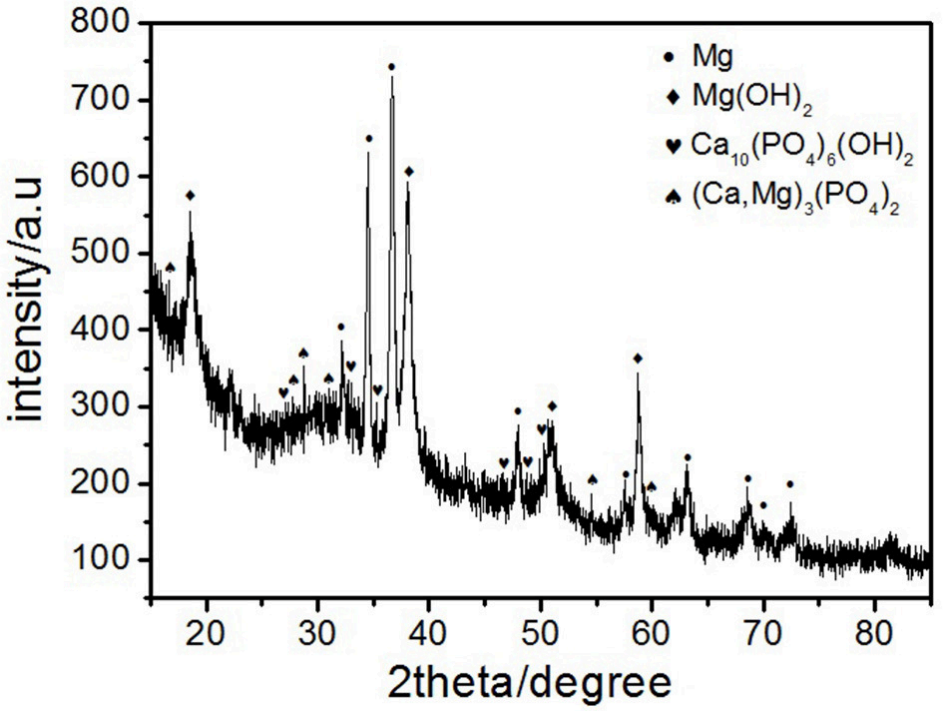
X-ray diffraction patterns of the corrosion products of as-cast alloy after immersion in SBF for 120 h.

Where ρ is the alloys density (g/cm^3^) (Shi et al., 2010). Figure 10 depicts the loss rates for the studied samples after 120 h immersions. Obviously, the extruded alloys own better resistances to the SBF compared to the as-cast one. With the increase of the extrusion temperature, the rate first dropped, reached the minimum at E350, and increased afterwards. The corrosion products and alloy surface morphology after immersion were studied through the SEM and EDS. As shown in Figures 11A–F, all immersed alloys have similar microstructures composed of white loose corrosion (e.g., Point A in Figure 11B) and tightly bounded products (e.g., Point B in Figure 11B). Irregular cracks are due to the loss of moisture in air during the drying process. The element analysis of the sample E330 shows that the corrosion products at A and B are composed of O, Mg, P, and Ca elements. The proportion of the quality of the elements is relatively similar, as given in Table 2. The crystal structures of corrosion products were determined through XRD. Result in Figure 12 proves the existence of the main composites of Mg, Mg(OH)_2_, Ca_10_(PO_4_)_6_(OH)_2_, and (Ca, Mg)_3_(PO4)_2_. During the immersion, OH^−^, Ca^2+^, H_2_${\text{PO}}_{4}^{-}$, and ${\text{HPO}}_{4}^{2-}$ in the SBF reacted with magnesium. The product hydroxyapatite (Ca_10_(PO_4_)_6_(OH)_2_) particles can accelerate the bone healings in the human body (Zhang et al., 2012c). The as-cast and extruded alloys possess good biocompatibility.

**Table 2:** EDS results of the E330 alloys after immersion in SBF for 120 h (wt.%).

**Position**	**O**	**Mg**	**P**	**Ca**
A	37.68	23.05	15.44	23.83
B	40.7	19.43	15.42	24.5

**Figure 13:**
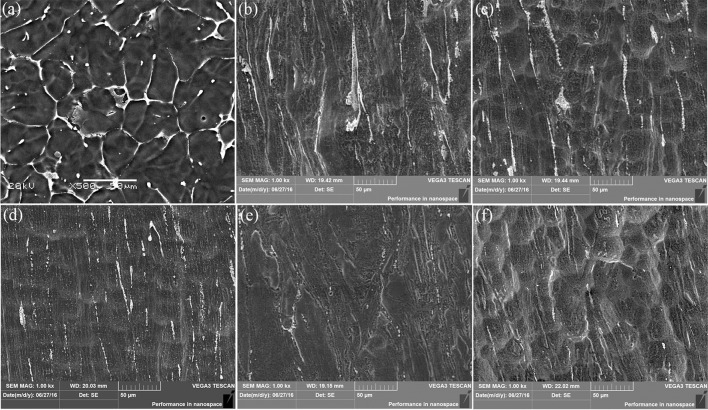
SEM micrographs showing the corroded Mg-2.0Zn-0.5Zr-3.0Gd samples after immersion for 120 h and corrosion product removal: **(a)** As-cast; **(b)** E330; **(c)** E340; **(d)** E350; **(e)** E360; **(f)** E370.

Despite similar biocompatibility, anticorrosive abilities differ from the as-cast and extruded samples. Figure 13 shows the surface morphology of the alloys after removing the etchant. For the as-cast alloy, it can be clearly seen from Figure 13a that the second phase at the microcrystal boundary acts as the cathode and the α-Mg matrix as the anode inside the grains. Different electrochemical potentials between the matrix and the second phase in the SBF will lead the solvation via electrochemical corrosion for the matrix but not for the second phase. The network shape of the phase, on the other hand, set up boundaries to prevent the extension of the corrosions. A careful check inside of the corroded matrix further shows the existences of the tiny holes, mainly due to the electrochemical corrosions next to small amount of the second phase dispersed inside of the matrix.

For the extruded alloy samples, the corrosion is uniform corrosion as shown in Figures 13b-f. This is attributed to the diminishing of differences between electrochemical potentials of the matrix and precipitations. During the extrusion, a large number of nanoscale rod-like (Mg, Zn)_3_Gd and granular Mg_2_Zn_11_ were precipitated, and uniformly distributed in the magnesium matrix as shown in Figure 8. A certain amount of alloying elements merged into the matrix, increasing the electrochemical potential of the α-Mg, and reducing the difference correspondingly. The potential difference becomes smaller between the second phase and the matrix of the α-Mg. During the immersion, the α-Mg anode was slowly dissolved, resulting in uniform corrosion. This discovery is in line with the previous research where the Mg_2_Zn_11_ phase can enhance the anticorrosive properties of the Mg alloys (Byun et al., 2013).

The surface morphology of the corroded alloys also follows the trend given by the extrusion temperature. Again, this is due to the temperature impacts on the microstructural changes. E350 has the best uniformity. This is not surprising because it owns the most abundant and best distributed DRXed grains, and a small amount of deformation of these grains. Taking the surface properties of another 4 immersed samples into consideration, it is generally found that the corrosion uniformity follows the ratio of the DRXed grains to the large deformed grains. A bigger ratio leads to a better uniformity.

**Figure 14:**
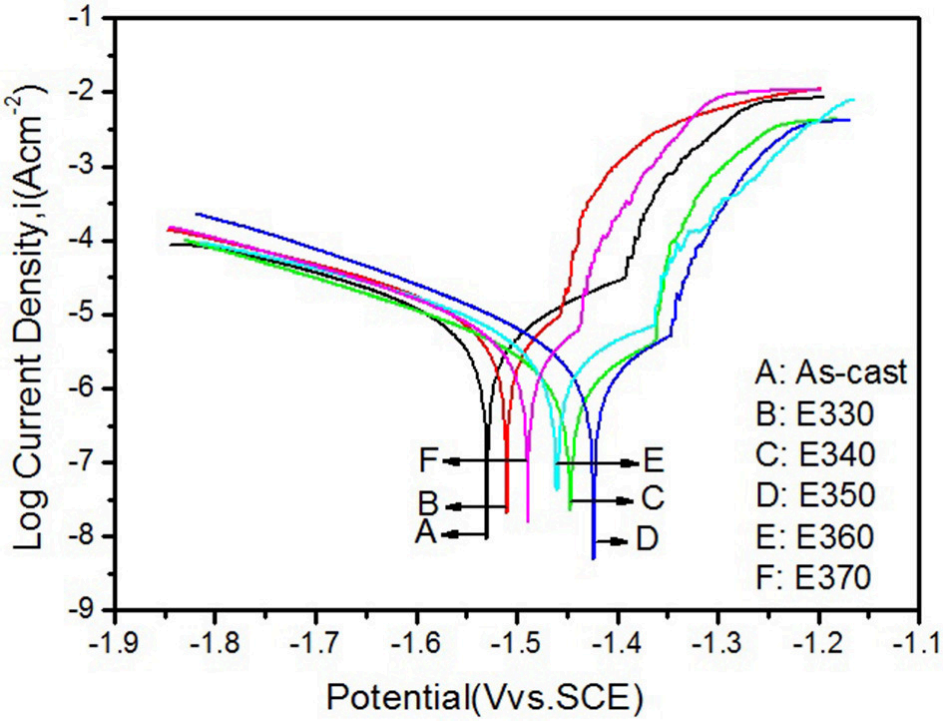
Potentiodynamic polarization curves of the Mg-2.0Zn-0.5Zr-3.0Gd alloys.

To crosscheck the immersion result, we analyzed the potentiodynamic polarization curves of the Mg-2.0Zn-0.5Zr-3.0Gd alloys performed on the electrochemical workstation. The curves were depicted in Figure 14 for the extruded samples and the as-cast one, and corresponding potential E_corr_ and current I_corr_ were listed in Table 3. Following the Tafel algorithm (Choi et al., 2012), the corrosion rates (P_i_) were also calculated and listed in the table. From the figure and table, it is obvious that the self-corrosion potentials are all above the original value of the as-cast, denoting smaller corrosive speed after extrusion. The extrusion temperature yields increase of the potentials for E330-E350, and then retreat of the values from E350-E370. Correspondingly, the anticorrosive properties were enhanced, and then weakened. The best performance is found for the E350 sample with the parameters of E_corr_ = −1.423 ± 0.003 V, I_corr_ = 3.226 ± 0.005 uAcm^−2^ and the P_i_ of 0.146 ± 0.003 mm/y. Physical origins for the potentiodynamic results are similar to the one given by immersion results, where relative larger amount of the DRXed grains offer smaller electrochemical potential differences once they were evenly merged into the matrix, and increase the potentials. The refined grains can enhance the alloy passivity by introducing smaller potential gradient into the system (Argade et al., 2012).

**Table 3:** Values measured from potentiodynamic and P_i_ of Mg-2.0Zn-0.5Zr-3.0Gd alloys.

**Material**	**As-cast**	**E330**	**E340**	**E350**	**E360**	**E370**
E_Corr_(Vvs.SCE)	−1.530 ± 0.004	−1.507 ± 0.003	−1.448 ± 0.003	−1.423 ± 0.003	−1.478 ± 0.002	−1.49 ± 0.004
I_Corr_(uAcm^−2^)	7.686 ± 0.006	5.662 ± 0.004	3.507 ± 0.004	3.226 ± 0.005	4.031 ± 0.003	4.629 ± 0.005
P_i_ (mm/y)	0.343 ± 0.002	0.255 ± 0.003	0.158 ± 0.003	0.146 ± 0.003	0.182 ± 0.002	0.209 ± 0.003

Despite the consistency of corrosion tests, higher static corrosion rates were noticed in the immersion test than these given by the Tafel algorithm. This is mainly arisen from the corrosive processes of the biometals. In the SBF, corrosion debuts from a passivation stage where the rate is rather low. At the middle stage, the rate increases due to the occurrence of electrochemical corrosion. After longer soaking time, the corroded layer gradually becomes thicker and prevent further chemical reactions. The rate becomes smaller, and the alloy tends to stable in the SBF (Abidin et al., 2011). The polarization curves mainly serve the rates at the first stage, and lead to smaller values compared to these given by static immersion tests.

We also evaluated the biosafety of the present implanting materials inside of human body. When the extrusion temperature is 350°C, the average dissolution of Gd in the first 120 h is about 0.0067 mg/cm^2^/d in the SBF. Assuming the exposing area is about 50~100 cm^2^ of the implant, a total daily release of the Gd is calculated to 0.335~0.67 mg. This number is much lower than 4.2 mg of daily absorption rate of rare earth elements in human body (Chen et al., 2014), indicating the total absorption of the released Gd^3+^. Typically, 10 g of the alloys is needed for the bone fracture fixation materials (Heublein et al., 2003; Seitz et al., 2016). Suppose a total degradation of the alloy implanted in a 70 kg human body happened, the LD50 level is calculated to 0.027 mmol/kg, much less than the Gd LD50 safe criterion of 0.1~0.3 mmol/kg in human (Bousquet et al., 1988). Thus, the alloy is safe.

## Conclusion

In conclusion, we have successfully tailored the microstructural and anticorrosive properties of the Mg-2.0Zn-0.5Zr-3.0Gd (wt.%) alloy by tuning the extrusion temperature. The network of the fish-bone shaped, ellipsoidal (Mg, Zn)_3_Gd phases, and lamellar LPSO phases in the as-cast Mg-2.0Zn-0.5Zr-3.0Gd (wt.%) alloy are modified to unDRXed deformed grains and fine DRXed grains. The area fraction of DRXed microstructure increased with extrusion temperature at the 330–350°C range but decreased at the 350–370°C range. The large sized secondary phases in the as-cast alloy were broken into particles with submicron size distributed at the grain boundaries in the E330 sample, while the secondary phase of the E350 sample was composed of rod-like (Mg, Zn)_3_Gd grains parallel to the extrusion direction and granular Mg_2_Zn_11_ phase. Along with their good biocompatibility, the as-extruded alloys own better anticorrosive performances in the SBF compared to the as-cast counterpart, with the one extruded at 350°C topping. Enhancement of the resistivity is attributed to the DRXed grains and their distributions in the matrix after extrusion in proper temperature, where electrochemical potentials of the biometallic systems can be modified accordingly.

## Author contributions

HY participated in the design of the manuscript and drafted the manuscript. J-BW analyzed experimental results. YX and YL carried out experiments. MH revised the manuscript.

### Conflict of interest statement

The authors declare that the research was conducted in the absence of any commercial or financial relationships that could be construed as a potential conflict of interest.
